# Investigation into problems associated with the endotoxin activity assay

**DOI:** 10.1186/cc10373

**Published:** 2011-10-27

**Authors:** N Matsumoto, G Takahashi, M Kojika, Y Ishibe, S Tatsuyori, S Yasushi, SK Inada, S Endo

**Affiliations:** 1 Department of Critical Care Medicine, School of Medicine, Iwate Medical University, Morioka, Japan

## Introduction

Endotoxin activity assay (EAA) levels were compared with endotoxin levels determined by the turbidimetric kinetic method.

## Methods

A specific method for the measurement of endotoxin, in the blood of patients under various conditions, and the influence of steroids on EAA levels and contamination of tubes used for the measurements were investigated.

## Results

EAA levels increased in patients with injuries and acute pancreatitis. EAA levels did not increase in patients infected with Gram-positive bacteria. Endotoxin levels determined by the turbidimetric kinetic method did not increase in patients with injuries and acute pancreatitis and Gram-positive bacteria. When patients with long-term steroid use developed shock due to infection with Gram-negative bacteria, EAA levels did not increase but endotoxin levels determined by the turbidimetric kinetic method increased. EAA levels, but not endotoxin levels determined by the turbidimetric kinetic method, were suppressed by giving steroids *in vitro*. Endotoxin was detected in the tubes used for the measurements. This was suppressed by the addition of polymyxin B and anti-factor C antibody. EAA levels tended to increase immediately after direct hemoperfusion using a polymyxin-B-immobilized fiber column (PMX-DHP).

## Conclusion

Our findings suggest that the EAA had limitations as a method to measure endotoxin.

